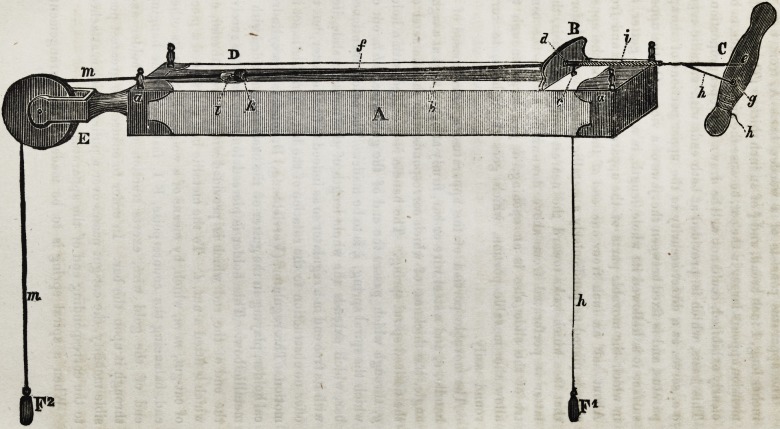# Description of a New Apparatus for Making Spiral Springs for Double Sets of Artificial Teeth

**Published:** 1854-07

**Authors:** C. N. L. Schmedicke

**Affiliations:** Practical Dentist, &c., in Berlin, Prussia.


### ARTICLE XI.

*Description of a New Apparatus for Making Spiral Springs for Double Sets of Artificial Teeth.* By C. N. L. SCHMEDICKE, Practical Dentist, &c., in Berlin, Prussia.

IN the following article, I take the liberty of laying before the respected readers of this journal, a new apparatus for making spiral springs for artificial teeth, which I have employed for some time with the best results, and which seems to me not unworthy a more extended use.

A
B
C
D
E
F1
F2
a
b
c
d
e
f
g
h
i
k
l
m

The apparatus consists of a wooden basis A, from 6 to 9 inches long, 1 inch broad, and $\frac{3}{4}$ of an inch thick. (The latter dimension appears larger in the cut, because the upper surface, to be clearly exhibited, could not well be drawn in proper proportion.) This basis, which is provided at both ends with metallic cups, *a a*, serves as a direct security to the upper portions of the apparatus, and is fixed between the jaws of a vice. On the upper surface it is hollowed its whole length, in a roundish gutter, *b*, in which the movable parts of the apparatus plays to and fro. About half an inch from one end of the basis, on the upper side, is placed perpendicularly a metallic slide, B. Just before that face which looks toward the nearest end of the basis, the latter is perforated from above downwards, *c*. In the centre of the slide also is an opening, *d*, just large enough to allow the movable portion, which goes through it, to turn round easily.

The movable portion of the apparatus, *c*, is composed of a handle, *e*, and a steel wire or bar, firmly attached to it at a right angle, the thickness of which corresponds with the diameter of the desired spiral spring. The handle is provided with a hole, *g*, through which passes the end of the gold wire, *h h h*, out of which the spiral spring, *i*, is to be made. The before mentioned bar, which extends the whole length of the metallic slide, lies with its free end in a regulator so as indeed to fix the latter, but to offer no obstruction to the rotation of the bar when this is set in motion. This regulator, (Vorrichtung,) D, consists of a cylindrical holder, playing in the gutter of the basis and provided with a metallic bow. This holder is pierced longitudinally to receive the end of the rod which is provided with a screw thread, on which is fitted a nut, *l*. To the metallic bow is attached a piece of cat-gut, *m m*, which, by means of a weight, F 2, on its other end, balancing the counterpoise, F 1, fastened to the hanging end of the gold wire, exerts traction upon the holder and through it upon the bar. In order to ensure the greatest possible mobility, the cat-gut passes over a pulley which is attached to the corresponding end of the apparatus.

When a spiral spring is to be made by this arrangement, the basis is securely fastened in a vice; one end of the gold wire is drawn through the hole, *g*, in the handle, and the other end is allowed to hang through the perpendicular opening, *c*. To this hanging end of the wire is attached a weight, F 1, and another, F 2, is made fast to the end of the cat-gut which runs over the pulley. When the movable part of the apparatus, the bar, is drawn by the operator towards himself, by means of the handle, the gold wire forms itself into spiral coils upon the bar. In this manner can be made a continuous spiral spring of great length, which can afterwards be divided in pieces to suit any particular set. When the gold wire is all worked up, the weights, F 1, F 2, are taken off, the nut, *l*, removed from the free end of the bar, which is then drawn out of the holder and the slide. The end of the gold wire, which was fastened to the handle, is thus cut off and the bar drawn out of the finished spring.

The spiral springs made by the aid of this apparatus, exhibit not only a high degree of elasticity, but also a great evenness in the coils; advantages which are due to the double traction of the weights and the equality of their motion. While the spiral coils of the gold wire are forming on the rod in consequence of the motion of the handle, they are drawn so strongly against the slide by the traction of the weight, F 2, that they are pressed against one another with the greatest closeness. On the other hand, the opposite traction of the weight, F 1, attached to the free end of the gold wire, which balances the other with mathematical accuracy, secures a uniformity in the single turns which leaves nothing to be desired. To these peculiarities the apparatus owes its utility.

## Figures and Tables

**Figure f1:**